# The Role of Selected Trace Elements in Oxidoreductive Homeostasis in Patients with Thyroid Diseases

**DOI:** 10.3390/ijms24054840

**Published:** 2023-03-02

**Authors:** Marcin Wróblewski, Joanna Wróblewska, Jarosław Nuszkiewicz, Marta Pawłowska, Roland Wesołowski, Alina Woźniak

**Affiliations:** Department of Medical Biology and Biochemistry, Collegium Medicum, Nicolaus Copernicus University, 85-092 Bydgoszcz, Poland

**Keywords:** oxidative stress, trace elements, thyroid diseases

## Abstract

Impaired levels of selenium (Se), zinc (Zn), copper (Cu), iron (Fe), manganese (Mn) and iodine (I) in the organism may adversely affect the thyroid endocrine system. These trace elements play a role in the fight against oxidative stress as components of enzymes. Oxidative–antioxidant imbalance is considered a possible factor in many pathological conditions, including various thyroid diseases. In the available literature, there are few scientific studies showing a direct correlation of the effect of supplementation of trace elements on slowing down or preventing the occurrence of thyroid diseases in combination with the improvement of the antioxidant profile, or through the action of these elements as antioxidants. Among the available studies, it has been shown that an increase in lipid peroxidation levels and a decrease in the overall antioxidant defense status occur during such thyroid diseases as thyroid cancer, Hashimoto’s thyroiditis and dysthyroidism. In studies in which trace elements were supplemented, the following were observed: a decrease in the level of malondialdehyde after supplementation with Zn during hypothyroidism and reduction in the malondialdehyde level after Se supplementation with a simultaneous increase in the total activity status and activity of antioxidant defense enzymes in the course of autoimmune thyroiditis. This systematic review aimed to present the current state of knowledge about the relationship between trace elements and thyroid diseases in terms of oxidoreductive homeostasis.

## 1. Introduction

Thyroid diseases can result from either the over or under-secretion of the thyroid hormones [1]. About 200 million cases of thyroid disease are diagnosed worldwide, while it is estimated that more than 1.5 billion people worldwide are at risk for thyroid dysfunction [2,3,4,5,6]. Numerous diseases can be caused by thyroid gland dysfunction, such as hypothyroidism, hyperthyroidism, Grave’s disease and Hashimoto’s disease [7,8]. Most trace elements are metals. Some of them are essential for the proper functioning of the body, while many others have harmful effects. Among trace elements, there are those that are essential, e.g., Cr, Cu, Se, Mo, I and Zn; probably essential, e.g., Mn, Si, B, Ni and V and potentially toxic, e.g., F, Pb, Hg, Cd, Al, As, Sn and Li [9]. Trace elements constitute an important structural component of thyroid hormones and are important for the metabolism and function of the thyroid gland itself [10]. Proper thyroid function depends on a variety of trace elements needed for hormone synthesis and metabolism. There is a dynamic balance between these elements [7,10,11,12,13,14,15,16]. Many studies on the importance of trace element imbalance in the body in the diagnosis and occurrence of various diseases have led the researchers to become interested in the biomonitoring of trace elements in human body fluids [17]. Trace element homeostasis and tissue concentration depend on the trace element uptake, compartmentalization, retention and clearance. However, more than the proper trace element availability in the environment and normal absorption and distribution to different tissues may be required to ensure correct cell function when abnormalities of mechanisms involved in the trace element cellular metabolism are present [18]. Determining trace element contents in biological fluids and tissues is essential due to their recognized role in several biochemical processes [19]. Essential trace elements should be equally monitored in the population due to their adverse effects after increased exposure [20]. The thyroid gland is an organ associated with the production of three hormones: calcitonin, 3,5,3′,5′-tetraiodothyronine or thyroxine (T4) and 3,5,3′-triiodothyronine (T3) [5]. Environmental factors such as impaired levels of selenium (Se), zinc (Zn), copper (Cu), iron (Fe), manganese (Mn) and iodine (I) may adversely affect the thyroid hormone system [21,22,23]. Iodine function in the human body is related to the proper functioning of the thyroid gland. Iodine is found in the chemical composition of T3 and T4 [24,25] and it also acts as an antioxidant directly or indirectly by inducing antioxidant enzymes [22]. Thyroid dysfunction is closely related to the adverse effects of reactive oxygen species (ROS), which are formed essentially by the reactivity of hydrogen peroxide (H_2_O_2_) [21]. Selenium, Cu, Zn and Mn are involved in protecting the organism against the effects of oxidative stress. Selenium is a component of enzymes associated with thyroid hormone balance, i.e., selenoproteins (SePs), such as glutathione peroxidase (GPx) [23,26,27,28]. Zinc and Cu play a key role both in the metabolism of I and thyroid hormones [21,29]. Copper assists the thyroid gland in producing T4 [21,30], while Mn affects the activity of deiodinase, and in combination with dopamine can indirectly modulate the secretion of thyroid-stimulating hormone (thyrotropin, TSH) secretion [21,31]. These two trace elements are also part of superoxide dismutases (MnSOD, Cu/ZnSOD) [26,29,32]. Iron is a component of the prosthetic group of catalase (CAT), which breaks down H_2_O_2_ [33]. Supplementation with I, Se, Mn, Zn and Fe may reduce oxidative damage by decreasing ROS production [21,22,26,34,35,36,37]. The aim of the present paper is to demonstrate the relationship between the aforementioned trace elements and markers of oxidative stress and the indicators of thyroid hormonal disorders.

## 2. Methods

We conducted an extensive literature search using the ISI Web of Science/PubMed/Science Direct/Google Scholar database for information on the influence of trace elements on oxidative stress in the course of thyroid diseases. The following keywords were used in data retrieval: (“thyroid gland” and “trace elements”, “iodine”, “selenium”, “zinc”, “copper”, “iron”, “manganese”); (“oxidative stress” and “thyroid disease” and “radioactive iodine”, or “iodine”, “selenium”, “zinc”, “copper”, “iron”, “manganese”); (“oxidative stress” and “iodine”, “selenium”, “zinc”, “copper”, “iron”, “manganese”); (“reactive oxygen species” and “thyroid disease” or “thyroid disorder”). There were no restrictions in collecting the data. No language restrictions were applied during the analysis. Rather, we tried to select articles from the last 20 years. After searching, we further examined the full text of the literature to determine eligibility for inclusion in this review. Editorials, conference abstracts and studies with incomplete or unavailable data were excluded.

## 3. Trace Elements and Thyroid Function

The thyroid gland has various functions and is an important endocrine gland in the human body [15]. Normal thyroid function depends on the presence of essential trace elements. Of these, iodine is involved in the pathways of thyroid hormone synthesis and metabolism [38]. These hormones, such as T4 and T3, are vital for various metabolic functions of human body tissues [15]. The thyroid gland is susceptible to trace elements because it can accumulate them for an extended period. Therefore, endocrine disruption may occur due to prolonged exposure to the high content of essential and toxic metals [39]. Five trace elements play critical roles in maintaining the physiological metabolism of the thyroid gland, namely I, Se, Zn, Mn and Cu [38]. The majority of the data that reflect the role of essential trace elements in thyroid function are empirical [38].

Selenium is essential in normal thyroid hormone metabolism and regulation as the integral component of iodothyronine deiodinases and is essential for many biological processes [35,40]. The thyroid is an organ with the greatest content of Se per tissue unit [13]. Selenium is a component of selenocysteine-containing SePs, including the iodothyronine deiodinases and GPxs which protect thyrocytes from oxidative damage. Low serum Se concentrations have been associated with hypothyroidism and autoimmune thyroid disease [41]. Maintaining a physiological concentration of Se within an optimum range is essential to ensure normal thyroid function and subsequent production of key regulators important for metabolism [42].

In turn, Zn is essential for the proper function of the type 1 5′-deiodinase enzyme, which catalyzes the conversion of T4 to its active form, T3, and decreases the metabolic rate [40,43]. Moreover, Zn deficiency inhibits thyrotropin-releasing hormone (TRH) synthesis. It is worth noting that hypothyroidism causes Zn deficiency in humans, and conversely, Zn deficiency can result in hypothyroidism [43]. Zn is also necessary for the proper function of the thyroid transcription factor 2 (TTF-2), which stimulates the expression of the thyroglobulin and thyroperoxidase genes. Thus, Zn is important in the production of thyroid hormones since thyrotropin and thyroperoxidase are proteins involved in synthesizing T3 and T4 in the thyroid [44]. What is worth emphasizing is that the thyroid is considered to have an important role in Zn homeostasis. The connection between Zn and thyroid metabolism is based on the hypothesis that the nuclear T3 receptor contains zinc-binding protein. Thyroid hormones have an effect on Zn absorption and excretion [45].

Copper is the third most frequent trace element and helps the thyroid gland in hormone production and absorption [46]. Copper stimulates the production of T4 and prevents over-absorption of T4 in the blood cells by controlling the organism’s calcium levels. Furthermore, Cu is required for the synthesis of phospholipids, which are essential for the stimulation of TSH [43,46].

Iron affects thyroid function and autoimmunity [47]. Iron is essential for the thyroid gland and is needed for effective iodine utilization by the iron-dependent enzyme TPO. Iron is required to catalyze thyroid hormone synthesis through TPO, which is heme-dependent [48]. Adequate Fe levels are important for maintaining normal thyroid function [14]. Li et al. [49] observed that in pregnant women, a lower Fe level was associated with a higher TSH concentration. Additionally, iron deficiency (ID) can reduce the conversion of T4 to T3 by interfering with the activity of thyroxine deiodinase [47].

In turn, Mn participates in various physiological processes, playing the role of a cofactor for multiple enzymes including transferases, hydrolases, lyases, isomerases, ligases and oxidoreductases [12]. Manganese can affect thyroid hormone homeostasis and neurodevelopmental processes as a result of both direct dysregulation at the level of the thyroid gland and thyroid hormones or indirectly via alterations in the dopaminergic control of the thyroid gland and its hormones [50].

Iodine is a relevant component of thyroid hormones and a particularly critical nutrient for child development. Its deficiencies may impair thyroid hormone synthesis and hence affect physical, neurological and intellectual development [51]. Iodine is a substrate for the biosynthesis of thyroid hormones which influence the metabolism and affect the expression of genes controlling various physiological functions, such as the embryogenesis, growth and development of the organism [11]. Concentrations of TSH, thyroglobulin (TG) and free triiodothyronine (FT3) in serum are considered alternative indicators of iodine status [52]. It has been reported that I deficiency may cause an increase in TSH levels and/or thyroid volume [45]. Unfortunately, increased I consumption does not always results in a notable decrease in TSH concentration, which remains within the reference range. Likewise, the free thyroxine (FT4) level changes very slowly after I administration [52]. Kravchenko et al. [52] suggest that TG seems to be a more sensitive indicator of I status after repletion.

**Figure 1:**
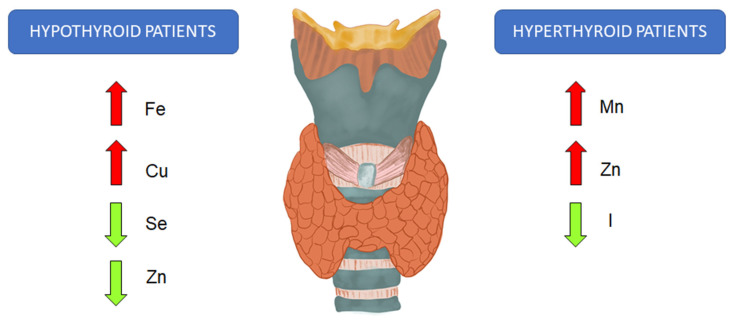
The changes in the levels of trace elements in the blood of patients with hyperthyroidism and hypothyroidism. Fe: iron; Cu: copper; Se: selenium; Zn: zinc; Mn: manganese; I: iodine.

The protective effects of I, Zn and Se on thyroid tissue as well as the antithyroid effects of toxic trace elements promoting a decrease in the levels of thyroid hormones and an imbalance of pituitary hormones (TSH) were detected [53]. The adequate status of the thyroid is determined by balanced consumption and accumulation of I and by the optimal trace elements level [53]. An imbalanced intake of trace elements other than I results in a higher risk of thyroid disease [40]. Low Se status has been associated with newly diagnosed Graves’ disease and Hashimoto’s thyroiditis [54]. On the other hand, low plasma Zn concentration has been associated with hypothyroidism and high concentration with hyperthyroidism (see Figure 1) [54]. It is speculated that Zn overload is a risk factor for benign and malignant tumors [55]. Zaichick [55] observed higher content of Zn in adenomatous thyroid tissue than in normal gland tissue. Moreover, he suggests that trace elements in thyroid tissue can be used as thyroid adenoma markers. Stojsavljević et al. [39] noted that trace element imbalance might be associated with the development of goiter and other thyroid diseases, including thyroid cancer and thyroid adenoma. They observed that the content of Mn, Cu, Zn and Se in pathological samples was significantly lower compared to the control. They also showed a higher Cu/Zn ratio in pathological thyroid samples than in the control ones. They indicated that this parameter might be considered an appropriate blood marker separating thyroid cancer patients from healthy subjects. Kazi Tani et al. [56] investigated Cu isotopes and the Zn/Cu ratio as possible biomarkers for thyroid cancer. They found a higher Cu/Zn ratio in thyroid cancer patients. Moreover, they observed lower δ^65^Cu (^65^Cu/^63^Cu ratio) plasma levels in thyroid cancer patients than in healthy controls, whereas thyroid tumor tissues presented high δ^65^Cu values. Al-Bazi et al. [7] suggest that hypothyroid patients appear to have an actual Cu level increase, whereas hyperthyroid patients have a deficiency of Cu. They also observed increased urinary Cu concentration in hypothyroid patients compared to the control healthy subjects and a significant positive association between Cu concentrations and thyroid disease. Likewise, Hanif et al. [17] compared levels of trace elements in the serum of hypothyroid patients, hyperthyroid patients and controls. They also observed elevated concentrations of Cu in hypothyroid patients and increased concentrations of Mn in hyperthyroid patients compared to controls (see Figure 1). Moreover, Hanif et al. [17] found some correlations between the levels of various metals (not discussed in this paper), while Zn was not significantly correlated with any other metals, which shows its independent variation in the serum of hyperthyroid patients. The pathogenesis of thyroid diseases is considered multifactorial, with particular emphasis on the role of environmental factors as disease inducers. The thyroid gland can accumulate metals for a long time. However, the content of metals in the gland varies depending on the application and their supply in the diet (food and water). Stojsavljević et al. [20] found differences in metal content regarding thyroid diseases such as a benign tumor, Hashimoto’s thyroiditis, multinodular goiter and thyroid cancer. They found the difference in metallic profile according to biological variables and demonstrated that each thyroid disease has its own unique metallic profile. Differences in the content of trace elements in various diseases suggest their participation in the etiology of thyroid diseases. However, different populations are exposed to different levels of metals in the environment. Thus, a single disease-specific trace element profile cannot be determined as different populations have dissimilar metal profiles [38].

Bibi and Shah [19] observed higher levels of Cu and Mn in the blood of thyroid cancer patients. They suggest that the disruption in the balance of essential (and toxic) trace elements in the blood may indicate the development and progression of thyroid malignancy [19]. The chemoprotective effect of Se on thyroid cancer is related to its ability to induce apoptosis, inhibit tumor growth and further tumor spread. However, the action of Se is not independent and is mediated by SePs. Decreased Se concentrations were reported to lead to developing thyroid cancer [38].

## 4. Reactive Oxygen Species in Thyroid Disorders

The term ROS refers to a wide variety of oxidant molecules of various properties and biological functions [57]. The role of ROS is not unequivocal. An increase in their concentration can induce oxidative stress [58,59]. When produced in a controlled manner they regulate cell signaling [60].

The role of ROS generated, among others, in mitochondria was analyzed in thyroid cancer metabolism. The authors suggest that disruption of mitochondrial oxidative phosphorylation and a correlated higher glucose and glutamine consumption creates an environment promoting cancer progression [61]. Another important source of ROS in cells are enzymes of the NADPH oxidases (NOX) of the family. There are three NADPH oxidases in thyroid cells: NADPH oxidase 4 (NOX4), dual oxidase 1 (DUOX1) and dual oxidase 2 (DUOX2) [58]. DUOX1 and DUOX2 generate H_2_O_2_ indispensable for the synthesis of thyroid hormones [62]. It has been shown that mutation of the gene *DUOX2* or *DUOXA2* (coding *DUOX* maturation or activation factors) through decreased generation of H_2_O_2_ leads to hypothyroidism [63]. Other studies also associate decreased generation of H_2_O_2_ with congenital hypothyroidism [64]. Dysregulated H_2_O_2_ metabolism, which can occur as a result of mutations in the *DUOX2* gene, is also observed in thyroid cancer. In familial non-medullary thyroid cancer (FNMTC), for example, the Y1203H germline *DUOX2* mutation was identified, accompanied by increased H_2_O_2_ generation [65]. It was attempted to explain the occurrence of myxedematous cretinism and thyroiditis by the increased generation of H_2_O_2_ and/or reduced ability to remove this ROS. The intracellular ROS-generating system in human thyrocytes also forms NADPH oxidase 4 (NOX4). Together with p22(phox) protein, it creates a heterodimeric enzyme complex (NOX4-p22(phox)), which, due to its intracellular occurrence, can participate in cytoplasmic redox signaling [66]. Increased content of these complexes in papillary and follicular thyroid carcinomas is associated with increased tumor cell proliferation and tumor progression [66].

Although H_2_O_2_ is essential for the synthesis of thyroid hormones, it is at the same time as toxic to thyrocytes as to other cell types [64]. Oxidant–antioxidant balance disorder in thyroid cells can lead to abnormal functioning of these cells and thyroid diseases [63,67,68]. There are mechanisms in eukaryotic cells that protect against excessive ROS generation. Among others, antioxidant enzymes, including CAT, SOD and GPx, as well as non-enzymatic scavengers, are involved in these processes. SOD in the dismutation reaction removes the superoxide radical, leading to the production of H_2_O_2_. CAT breaks down H_2_O_2_ to water and molecular oxygen [69,70]. GPx (family of multiple isozymes) reduces H_2_O_2_ to water using reduced glutathione (GSH) as an electron donor [71,72]. Eight GPxs (GPx1-GPx8) have been identified in mammals so far, five of which contain selenium (GPx1-4 and 6) [73,74]. Thyroid cells also protect themselves against oxidative damage using SOD, CAT and GSH [75]. However, a study in Wistar rats showed that, compared to other organs, the thyroid gland is characterized by low SOD and CAT activity. In contrast, high activity was noted for GPx and glutathione reductase (GR) [76]. Against the high amounts of H_2_O_2_, thyroid cells are protected by eleven SePs [77], mainly including GPx3 [16]. In vitro studies have also shown (in rat thyroid FRTL-5 cells) that peroxiredoxins (Prxs) 1 and 2 are involved in the removal of H_2_O_2_ generated in thyroid cells under the influence of TSHs [75]. Gérard et al. [78] have shown Prx 5 expression in the human thyroid gland participating in the reduction of peroxides. Enhanced activity of this enzyme was observed in human thyroid specimens from patients with Graves’ disease [78].

Since virtually all body tissues are influenced by thyroid hormones, disruption of their secretion can affect the systemic oxidant–antioxidant balance. It has been shown that thyroid hormones elicit ROS generation, which leads to oxidative stress [79,80]. This is mainly due to the fact that these hormones accelerate basal metabolism in cells [81]. However, in vitro studies have shown that thyroid hormones exhibit ROS scavenging capacities [82]. Correlations between thyroid hormone levels and ROS generation and removal are quite complex. Even contradictory research results are often obtained, but the trend is that hyperthyroidism exacerbates oxidative stress, whereas hypothyroidism results in nondetectable-to-mild oxidative stress [81]. In venous blood, for example, higher antioxidant enzyme activity is observed in patients with papillary thyroid carcinoma and Hashimoto’s thyroiditis than in healthy controls [83].

## 5. Impact of Trace Elements on Oxidative Stress

Trace elements participate in the regulation of homeostasis on many levels [84,85]. Selenium, Zn, Cu, Fe, Mn and I are particularly important in maintaining of oxidoreductive balance [86,87,88,89,90,91]. Trace elements are directly and indirectly involved in generating of ROS, promoting pro-oxidative processes [89]. On the other hand, trace elements, especially in the form of metalloproteins, reduce oxidative stress by promoting antioxidant metabolic pathways [92].

Selenium is an essential element not only for humans but also for all mammals [93]. Selenium in an inorganic form exists mainly as Se^4+^ and Se^6+^ [94]. In living organisms, this trace element is found in the form of SePs and has antioxidant properties [86]. Selenium is a component of two metabolically significant amino acids, namely selenomethionine and selenocysteine [95]. Both amino acids are formed by replacing the sulfur atom present in methionine and cysteine with a Se atom [96]. Selenocysteine is present in the active sites of enzymes with antioxidant properties such as GPxs and thioredoxin reductases [97,98,99,100]. Recent studies indicate the presence of 25 SeP genes in the human genome, and the role of these biomolecules is mainly related to the neutralization of ROS [101]. GPxs constitute a large group of enzymes belonging to the class of peroxidases and require the glutathione in the reaction environment for their activity [74]. Deficiency of Se in the diet leads to a decrease in the activity of GPxs [102]. Thioredoxin reductase is classified as a high molecular weight selenoenzyme [99]. This enzyme, together with NADPH and thioredoxin, constitutes the thioredoxin system with antioxidant properties [103]. The role of the thioredoxin system in counteracting oxidative stress is related to the reduction in peroxiredoxin proteins [104]. Peroxiredoxins neutralize H_2_O_2_ and, as a result of this reaction, undergo oxidation. Selenoprotein P (SePP) also influences the redox balance. This biomolecule, acting through its intrinsic thioredoxin domain, distributes Se necessary for synthesis of intracellular GPx and weakens the activation of the 5′ adenosine monophosphate-activated protein kinase (AMPK) pathway [105,106].

Zinc is a microelement necessary for the proper functioning of about 300 metalloenzymes from the groups of oxidoreductases, hydrolases and ligases [107]. Zinc ions are cofactors of enzymes and deficiencies of this element lead to a decrease in the activity of zinc-dependent enzymes [108]. Zinc has antioxidant properties. It directly prevents the formation of the hydroxyl radical (^•^OH) and superoxide radical (O_2_^•−^), competing with pro-oxidative metals such as Fe^2+^ or Cu^2+^ [109]. This trace element inhibits NADPH oxidase. One of the products of the reaction catalyzed by NADPH oxidase is O_2_^•−^ [110]. Under physiological conditions, Zn forms a complex with proteins belonging to the metallothionein family [111]. Metallothioneins are proteins rich in cysteine residues, capable of binding divalent trace elements, such as Zn, Cu, Cd, Hg and Pb [112]. In addition to binding pro-oxidant metals, due to the presence of cysteine in the protein structure, metallothioneins neutralize ROS [113].

Copper may occur in the form of Cu^+^ and Cu^2+^, which makes this element an excellent cofactor for many enzymes involved in electron transfer metabolic pathways [114]. Copper is a trace element with pro-oxidant and antioxidant properties [115,116]. In the Fenton-like copper redox reaction, ROS are formed. [117]. On the other hand, Cu and Zn are metals found in the active site of antioxidant enzymes such as SOD-1 and SOD-3 [118]. A significant part of the plasma Cu pool is built into a protein with antioxidant properties—ceruloplasmin [119]. This protein is classified as an oxidoreductase and its cofactors are Cu ions [120]. Ceruloplasmin is involved in Fe homeostasis by catalyzing the oxidation of Fe^2+^ to Fe^3+^, and reduces the generation of ^•^OH [121].

Iron not bound to proteins is highly toxic to cells and induces oxidative stress [122]. Under physiological conditions, almost all Fe is bound to proteins such as hemoglobin, myoglobin, transferrin, ferritin and hemosiderin [123]. In biological systems, Fe^2+^ ions and H_2_O_2_ undergo the Fenton reaction [124]. The products of this reaction are highly reactive ^•^OH and Fe^3+^ [125]. Free Fe is a source of ROS, while Fe bound to proteins has antioxidant properties. The heme group, containing Fe^3+^, is the catalytic center of all four polypeptide chains of CAT [126]. CAT is an enzyme from the oxidoreductases class that catalyzes the decomposition of H_2_O_2_ into water and oxygen [33].

**Figure 2:**
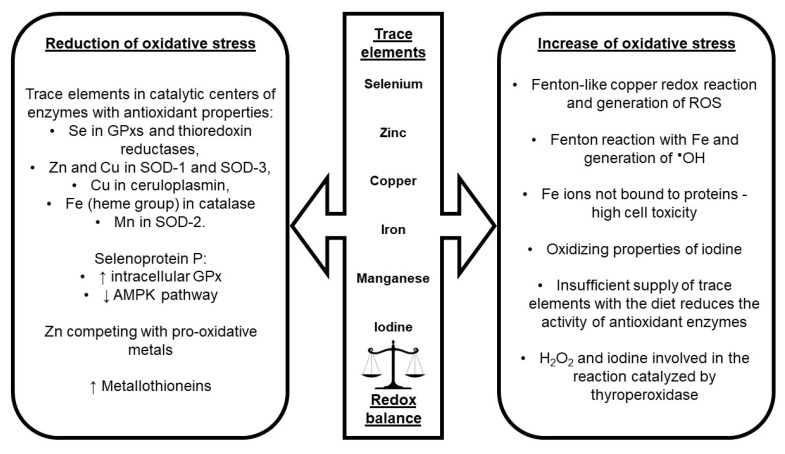
Mechanisms regulating pro-oxidant and antioxidant homeostasis via the trace elements. ^•^OH: hydroxyl radical; GPxs: glutathione peroxidases; H_2_O_2_: hydrogen peroxide; ROS: reactive oxygen species; SOD: superoxide dismutase.

Manganese, especially in the form of Mn^2+^ cations, is present in the active sites of many enzymes [127]. The mitochondrial form of SOD contains Mn and participates in the neutralization of ROS [90]. The SOD-2 isoform is particularly important for maintaining the redox homeostasis of the mitochondria, which are the site of the generation of numerous ROS. Iodine is a trace element that is not directly involved in redox balance. Studies have been conducted on I supplementation, that is by consuming salt enriched with iodate. Iodate is a strong oxidant and some research suggests that I ingestion may cause oxidative stress [91]. Thyroid hormones, by regulating metabolism, influence the generation of ROS and enhance oxidative stress [79]. ROS are necessary for the proper functioning of the thyroid gland. One of the steps in the synthesis of T3 and T4 is the oxidation of iodine with the participation of thyroperoxidase and H_2_O_2_ [128]. Maintaining an adequate supply of trace elements with the diet and exposure to their presence in the environment is significant for maintaining homeostasis between pro-oxidant and antioxidant metabolic pathways. Figure 2 shows the mechanisms regulating redox balance via the trace elements.

## 6. Speciation of Selected Trace Elements in Thyroid Diseases

Speciation of trace elements should be considered in two ways: (1) as elements occurring in body fluids and tissues and (2) as dietary components. In the body, the discussed trace elements, i.e., Se, Zn, Cu, Fe, Mn and I, can occur in various types of bonds. These include bonds based on covalent bonds and the coordination of ions at various oxidation states [129]. Selenium in the human body is mainly found in the form of a covalent compound as well as the amino acids selenomethionine and selenocysteine. In this amino acid form, it builds the proteins of important enzymes that ensure redox balance, e.g., GPx. Zn and Mn build metalloenzymes by binding Zn^2+^ and Mn^2+^ ions to a protein ligand (forming a metal–ligand complex). Analogous metal–ligand complexes are formed by Cu^2+^, Cu^+^, Fe^2+^ and Fe^3+^ ions. In hemoglobin, the protein forms a complex with Fe^2+^ ions. Copper is known to occur in the serum, among others, in the form of transport complexes with proteins. Although such complexes are formed by both Cu^+^ and Cu^2+^ ions, in order for Cu to be transported into cells, Cu^2+^ ions must first be reduced. The last of the discussed trace elements, I, forms covalent bonds with atoms in the form of organoiodine compounds, which include thyroid hormones T3 and T4 [21,34,130,131,132]. In studies on the effect of supplementation with various elements on oxidative stress parameters in thyroid diseases, Zn, Se and I in the form of inorganic compounds caused an increase in the level of antioxidant defense enzymes and a decrease in the level of oxidative stress parameters. Conversely, lowering the content of these elements resulted in lowering the level of antioxidant enzymes. An analogous effect was produced by the administration of selenious yeast, even though the bioavailability of Se from this supplement is greater than when inorganic Se compounds are used [34,130,133]. The authors of the discussed studies showed that in many cases the level and bioavailability of one element is influenced by another or other trace elements. Examples include Mn and Fe. When there is a low level of Fe in the blood, Mn, in the absence of competition, accumulates more, which does not mean that there will be more of it in terms of the level of enzymes with which it forms connections important from the point of view of homeostasis or the occurrence of diseases such as thyroid diseases [21]. Essential trace elements in body fluids as well as in tissues form complexes with proteins. Such examples are Fe complexed by transferrin or ferritin [134], Cu bound to ceruloplasmin or albumin [135,136] and Zn bound to albumin and α_2_-macroglobulin [137]. The combinations of these elements are more or less specific, which reflects on bioavailability and their ability to obtain a toxic form. For example, overloading the body with Fe results in the formation of a fraction of Fe not bound to transferrin responsible for participating in oxidative stress through the Fenton reaction. The contribution of speciation is, of course, indisputable in terms of disease pathogenesis, but it is also difficult to study because of the enormous potential for trace elements to bind to various proteins, sugars or nucleic acids in the body, binding in a specific/non-specific manner. Additionally important are interactions between trace elements, i.e., competitive binding to molecules. All this means that despite the knowledge of the speciation of trace elements, it is still impossible to predict the physiological or pathological effect of their action [129]. Nevertheless, any research is valuable in this aspect as well, as it brings us closer to a better understanding of the pathogenesis of many diseases, including thyroid diseases.

## 7. Impact of Trace Elements on Oxidative Stress Markers in Thyroid Diseases

Thyroid diseases lead to various pathological changes throughout the gland, including hormonal ones. These hormones may play an essential role in the defense against oxidative stress. This is possible through their impact on the antioxidant system. Balanced supplementation with trace elements can affect hormone levels and thus regulate the oxidant–antioxidant balance [81,138]. In the early stages of pregnancy, the development of the fetal central nervous system is completely dependent on the production of thyroxine in the mother’s body. Iodine deficiency is associated with pregnancy complications and can interfere with TSH production [37] and increase oxidative stress in the thyroid gland [130]. Optimal levels of I intake induce an improvement in antioxidant profile [22]. Oxidative metabolism increases during pregnancy due to the increased oxygen demand of the mother and fetus, thus inducing the production of ROS [37]. Assessment of urinary I levels allowed Restini et al. [37] to examine thyroid function and determine which I deficiency profile affects thyroid function and the biomarkers of oxidative stress in pregnant women (up to the 14th week of gestation). According to the researchers, iodine plays a significant role in antioxidant capacity during gestation. Normal urinary levels of I in pregnant women positively correlated with increased levels of α-tocopherol. Iodine deficiency did not affect the changes in TSH levels, nor produce autoantibodies against thyroglobulin (TgAb) and thyroperoxidase (TPOAb). Together with the increase in the value of advanced oxidation protein products (AOPP), I deficiency elicited exacerbation of the antioxidative profile in the subjects [37].

Treatment options for thyroid disorders include antithyroid drugs, radioactive I therapy and surgery. Radioactive iodine therapy (RAI, also called ^131^I), introduced as early as the 1970s, can be used to ablate (destroy) any thyroid tissue not removed via surgery or to treat some types of thyroid cancer [139]. Ionizing radiation (high doses) can cause ROS formation, peroxidation of cell membrane lipids, resulting in DNA damage and carcinogenesis [131,139]. Studies described in the work of Rosário et al. [140] in 40 thyroid cancer patients showed a significant increase in plasma levels of the oxidative stress biomarker, 8-epi-prostaglandin F2 alpha (8-epi-PGF2a). Oxidative stress persisted with high intensity several days after ^131^I administration. The researchers additionally showed that the use of supplementation of 2000 mg of vitamin C, 1000 mg of vitamin E and 400 µg of Se for 21 days prior to RAI in the 20-person intervention group significantly reduced the levels of 8-epi-PGF2a. They concluded that ^131^I ablation causes oxidative stress, which can be minimized by using antioxidants [140]. Wolfram et al. [141] assessed 8-epi-PGF2a levels in seventeen patients who received ^131^I therapy for hyperthyroidism and seven patients treated for differentiated thyroid cancer. RAI led to an increase in the concentration of 8-epi-PGF2, compared to the concentration of this parameter in the control group and with the pre-therapeutic values measured in patients. The researchers failed to show a correlation between the administered radioiodine dose and 8-epi-PGF2a levels; however, they suggested that 8-epi-PGF2a levels have a potential prognostic value in terms of assessing the extent of radiation damage. Enzymatic and non-enzymatic antioxidant systems are responsible for ROS scavenging and elimination [141]. In the study conducted by Konukoglu et al. [132] in 30 patients with papillary and follicular thyroid carcinoma, ^131^I-therapy has been shown to lead to increased lipid peroxidation, expressed as a significant increase in MDA.

Selenium is also an essential element responsible for the synthesis and function of thyroid hormones (iodothyronine selenodeiodinases), which catalyze extrathyroidal production of T3 [142]. Compared to the other organs, it is the thyroid gland that contains the highest concentration of this trace element [133]. The most common form of Se supplementation is sodium selenite [143]. Selenium as an antioxidant protects the thyroid gland from oxidative damage by controlling the number of free radicals and H_2_O_2_ [133]. The intrathyroidal content of this micronutrient is influenced by the intensity of oxidative stress and the daily diet [144]. O’Grady et al. [145] found no statistically significant correlations between increased Se intake and the possibility of the occurrence of thyroid cancer. Selenium deficiency is associated with the pathogenesis of autoimmune thyroiditis (AT), which is associated with a state of higher oxidative stress. Supplementation with this microelement may influence, to a varying degree, the decrease in TPOAb titer in connection with, among others, the organism demand for I [130]. Thyroid function does not always improve after the introduction of Se as an antioxidant supplementation. Impairment of the utilization and at the same time the effectiveness of Se may be associated with changes in the activity of the antioxidant enzyme known as GPx, which protects cells from ROS [130,144]. Changes in GPx activity in erythrocytes may be dependent on single-nucleotide polymorphism (selenoprotein single-nucleotide polymorphisms, SNPs) occurring in genes encoding individual SePs [130]. de Farias et al. [130] believe that the reduction in TPOAb levels may be a result of increased Se levels and improved redox status in thyrocytes occurring during supplementation. In patients with different GPx1 genotypes, no differences were noted in the decrease in TPOAb titer in response to a Se supplementation diet. The correlation between Se levels and GPx activity in children with congenital hypothyroidism was presented by Chanoine et al. [146]. The researchers measured plasma Se concentrations, GPx in erythrocytes and thyroid function parameters, before and after a 3-month supplementation. Selenium supplementation failed to correct thyroid hormone abnormalities (i.e., increased serum T3 levels and fT4/fT3 ratio). A negative correlation was found between baseline plasma Se concentrations and changes in GPx activity in erythrocytes, as the lower the Se concentration before supplementation, the greater the increase in GPx activity. Tian et al. [133] concluded that a three-month supplementation of Se alleviates the autoimmune process of the thyroid (lowers TPOAb) by improving the antioxidant status of the body. In their study, they assessed such parameters as MDA levels, antioxidant defense based on total antioxidant capacity (TAC) and SOD levels, and TSH, TgAb and TPOAb values. The degree of reduction in SOD levels was not proportional to the reduction in TgAb and TPOAb. There was a negative correlation between TAC and TgAb/TPOAb and a positive correlation between MDA and TgAb/TPOAb.

Important cofactors involved in the regulation of antioxidant enzyme activity, besides Se, are such trace elements as Cu, Zn and Mn. The levels, among others, of these antioxidants, concentrations of thyroid hormones such as TSH, FT3, FT4 and TPOAb, levels of SOD, GPx and GR activity, and TAC in patients with newly diagnosed thyroid dysfunction were determined by the team of Maouche et al. [21]. Nutritional supplementation with antioxidant trace elements and vitamins A, C and E was the basis for excluding patients from the project. Patients showed endocrine disorders related to levels of TSH, free thyroid hormones (FT3, FT4) and TPOAb, oxidative damage due to lack of antioxidant protection (SOD, GPx, trace elements) and chronic inflammation. All patients with thyroid disorders showed significant Se depletion, with a concomitant reduction in GPx activity, indicating a deficiency of SePs in thyroid tissue and a reduced antioxidant defense system. Iron deficiency in patients led to a greater accumulation of Mn. The researchers suggest that the heightened thyroid oxidative stress may result from the occurring Tf/Tf receptor system, and elevated Mn is produced via an extracellular Tf-manganese redox mechanism [21]. Among all thyroid dysfunction groups, participants with hyperthyroidism showed greater oxidative stress compared to patients with hypothyroidism. TAS levels were strongly reduced in study participants with hyperthyroidism, and to a lesser extent in patients with subclinical and overt hypothyroidism. Patients with hypothyroidism may be deficient in such microelements as Zn, Mg and vitamin A [34]. Rabbani et al. [34] examined the effects of supplementation with zinc gluconate, magnesium oxide and vitamin A on the serum levels of thyroid hormones such as TSH, FT3, FT4, total T4 (TT4) in 43 hypothyroid patients and 43 subjects of the placebo group. In addition, they assessed oxidative stress by examining MDA and TAC levels. Combined supplementation with Zn, Mg and vitamin A resulted in a significant increase in FT4 levels compared to the control group. However, the above intervention had no effect on serum TSH, FT3, TT4 and MDA levels. In addition, a significant reduction in serum TAC levels and a significant increase in CRP levels were observed in the placebo group. Low TAC levels may indicate oxidative stress or increased susceptibility to oxidative damage. The researchers concluded that supplementation led to an increase in antioxidant pathways, and the synergistic effect of using Zn, Mg and vitamin A may represent a new approach in antioxidant defense [34]. Changes in oxidant/antioxidant parameters, including SePP and the trace element status (urinary I, plasma Se and serum Zn levels) in 29 children (aged 8–16 years) with Hashimoto’s thyroiditis were reported in Sur et al. [23]. The researchers observed an impaired antioxidant defense system in hypothyroid patients (decreases in SOD and GPx activity). The significant role of Se for thyroid homeostasis was confirmed based on the positive correlation between SePP and fT3 in the group with Hashimoto’s thyroiditis. A decrease in plasma SePP was also documented in these patients, while plasma Se levels were not significantly different. Most of the examined children with Hashimoto’s thyroiditis were found to be deficient in I. The TPOAb levels were inversely correlated to the urinary level of I. Oxidative stress observed at I deficiency may be a contributing factor to thyroid dysfunction. The study and control groups had normal serum Zn levels, but children with Hashimoto’s thyroiditis had these levels significantly reduced compared to controls [23]. The correlation between Zu and Cu levels and indicators of oxidative stress in patients with thyroid disease varies. Studies presented by Szczepanik et al. [29] failed to show the correlation between Zn and Cu concentrations and oxidative stress indicators and the levels of antibodies (TPOAb, TgAb) indicating the occurrence of Hashimoto’s thyroiditis in female patients. On the other hand, patients with endemic iodine deficiency nodular colloidal goiter showed elevated Cu levels and reduced Zn levels in thyroid tissue associated with the occurrence of oxidative damage [147]. The available literature lacks scientific studies demonstrating links between Fe and indicators of oxidative stress caused by hyperthyroidism or hypothyroidism, except for thyroid cancer. 15-deoxy-Δ^12,14^-prostaglandin J_2_ (15d-PGJ_2_) induces apoptosis and inhibits growth of the thyroid papillary cancer cell line, CG3, through the generation of intracellular ROS. CAT, N-acetylcysteine and iron chelator desferrioxamine completely eliminated 15d-PGJ_2_-induced ROS generation and cytotoxicity in CG3 cell [148]. Table 1 summarizes the examined parameters of oxidative stress and trace metal concentration in the course of selected thyroid diseases.

## 8. Conclusions

Oxidative stress is a lack of balance between the level of oxidants and antioxidants, with a predominance of the former, which leads to the impairment of redox signaling and monitoring and/or molecular damage that alters cellular function. Redox imbalance plays a significant role in the pathogenesis of many diseases. Thyroid diseases are a public health problem worldwide. Both hypothyroidism and hyperthyroidism are associated with increased ROS generation, reduced antioxidant capacity and consequently increased oxidative stress. Trace elements may be involved in neutralizing ROS. Additionally, trace elements are essential for proper thyroid hormone metabolism, while deficiencies can impair thyroid function. A proper diet allows us to maintain proper hormonal balance, reduces the incidence of thyroid dysfunction and protects the body against oxidative stress. Based on the reports mentioned above, it seems reasonable to use the levels of trace elements to diagnose thyroid diseases. Trace element ratios also seem to be a helpful indicator in the assessment of thyroid disorders. Additionally, there are few scientific studies in the available literature showing a direct correlation of the impact of trace element supplementation on slowing or preventing the onset of thyroid disease with an improvement in the antioxidant profile, either through the direct action of these elements as antioxidants or their indirect action through the induction of antioxidant enzymes. Hence, further investigations are required.

## Figures and Tables

**Table 1:** Changes in the oxidative stress parameters and trace element concentration in patients with selected thyroid diseases.

Disease	Patients	Parameters	Refs
Hypothyroidism	n = 43, age (20–65)	Lower level of MDA and CRP after supplementation zinc gluconate, magnesium oxide and vitamin A; no significant change in level TAC	[34]
Autoimmune thyroiditis	n = 18, age (40.2 ± 10.9)	Lover level of MDA, higher level of TAC and SOD after supplementation selenious yeast	[133]
	n = 28, age (20–58)	Higher level of GPx after supplementation sodium selenite or selenomethionine vs. placebo group	[130]
Dysthyroidism	n = 170, age (30–50)	Lower concentration of Se and Cu and higher concentration of Mn vs. control group Lower level of GPx, mitochondrial SOD and TAS vs. control group	[21]
Hashiomoto’s thyroiditis	n = 42, age (45–60)	Higher level of TBARS; no significant differences in the concentration of Cu and Zn vs. control group	[29]
Thyroid cancer	n = 30, age (43 ± 7)	Higher level of MDA, lower level of GR and GPx vs. control group Higher level of MDA, GR and GPx after radioiodine therapy vs. control group	[132]

CRP: C-reactive protein; Cu: copper; GPx: glutathione peroxidase; GR: glutathione reductase; MDA: malondialdehyde; Mn: manganese; n: number of patients; Se: selenium; SOD: superoxide dismutase; TAC: total antioxidant capacity; TAS: total activity status; TBARS: thiobarbituric acid reactive substances; Zn: zinc.

## Data Availability

Not applicable.
